# Lead with speed: Recasting the forensic laboratory product line and service delivery model

**DOI:** 10.1016/j.fsisyn.2024.100471

**Published:** 2024-05-04

**Authors:** Ray A. Wickenheiser, Catherine M. Knutson

**Affiliations:** a New York State Police Crime Laboratory System, Albany, NY, USA; b Minnesota Bureau of Criminal Apprehension Forensic Science Services, St. Paul, MN, USA

**Keywords:** Investigative Leads, NIBIN, NDIS, CODIS, Business case, Return on investment, Forensic lead program

## Abstract

Forensic laboratories face a multitude of challenges when striving to deliver services to the criminal justice system. While many of these issues change over time, one in particular seems to endure the test of time … the need for faster results. Law enforcement wants and needs quicker response times to access critical information required to investigate their cases. One answer to this persistent problem is evolving technology. Technology not only permits a much quicker response than forensic laboratories are currently delivering, it can open the door to solving previously unsolvable cases. Along with applying new technology, an evaluation of current forensic laboratory product lines, service delivery models, and mindset regarding the role of forensic science-based investigative leads (termed forensic leads) is warranted. Resources and strategic planning are needed to realize the full potential of evolving technologies and what forensic laboratories can do to provide actionable and timely forensic leads to our criminal justice partners as a normal course of action instead of as an exception.

This proposal is to establish a permanent, designated Forensic Lead Program (FLP) that resides under the umbrella of an accredited forensic laboratory and is tasked with the development and release of forensic leads. The FLP involves a focused menu of services, defined personnel roles, strict protocols, short turnaround time, standardized expectations, and targeted training, combined with the sense of urgency needed for consistent delivery of timely and actionable forensic leads. A dedicated FLP will save time and money by providing critical information for more focused investigations. ‘Speed is the need’ … for quick identification of those that threaten public safety and for the equally quick elimination of those wrongfully accused.

Programs at two large state forensic laboratories will demonstrate how these concepts could be implemented along with their learning experiences. A business case will also be included to demonstrate the cost benefit of the Forensic Lead Program for DNA (CODIS – Combined DNA Index System) and NIBIN (National Integrated Ballistic Information Network), however other section services are expected to see similar benefits. Improving the response time by one day saves $1677.75 per $1 spent [1]. The return on investment (ROI) for applying DNA to firearms evidence returns $47.88 per $1 spent, or an 4,788 % ROI. Applying NIBIN (National Integrated Ballistic Information Network) to firearms evidence to provide investigative leads is $502.19 per $1 spent, which is a 50,219 % ROI. Recasting the forensic laboratory product line and service delivery model to ‘Lead with Speed’ makes both economic and investigative sense.

## 1 Introduction

Forensic analysis is frequently performed to the highest level of completeness permitted by validated laboratory methods with little to no differentiation between delivering forensic leads and courtroom ready results [2]. This current model as applied in the United States, where all analyses are completed to the same service level usually requires long training programs along with submission limitations and drawn-out implementation plans to increase capacity if laboratories are ever going meet the demands for their services. These challenges are particularly acute for multi-jurisdictional laboratories with multiple laboratory locations such as United States state government forensic laboratories. This is not a sustainable way to do business with existing resources. Meanwhile, investigators see opportunities to get answers to their questions and solve their cases with new technology. However, the laboratory is too busy with what is on its plate at any given time to validate yet another piece of new instrumentation. Instead, many forensic laboratories rely on strict case acceptance criteria to place restrictions on their services such as limiting the number of items submitted per case or the types of examinations provided. Hence the laboratory is frequently seen as the “bad guy” despite the justifiable reasons behind restrictive evidence submission and analysis rules. Investigative resources are burning while cases sit in the forensic laboratory awaiting testing. ‘Speed is the need’ for quick identification of individuals threatening public safety and for the equally quick elimination of those wrongfully accused.

Forensic laboratories inherently understand the value of faster forensic leads. This is evident by the practice of triaging and juggling cases when a rush request is received by the customer. However, this prioritization of critical cases is usually done by exception, which quickly disrupts the efficiency of the overall testing processes. Ideally, immediate attention to high priority cases and time sensitive lead generation would be integrated into the business operations through a thoughtful, deliberate and planned service delivery strategy.

For clarity, reference to a “forensic lead” in this paper refers to information generated through forensic testing that may assist in the furtherance of an investigation. While the term “forensic intelligence” is also commonly used in the context of investigative assistance through forensics, it is generally more expansive; going beyond individual forensic tests into data analytics, pattern recognition, and cross-case evaluation. However, evaluation of analytical data to the extent where case specific propositions are considered in forensic intelligence, is beyond the scope of the term forensic lead as applied in this paper [3].

Technology and process changes are an answer to deliver quicker response times. Rapid DNA analysis can be completed in under 2 hours and National Integrated Ballistic Information Network (NIBIN) programs are turning out leads in 24–48 hours [4]. Investigators see this and want it.

Some law enforcement agencies contemplate how they can purchase and implement the new technology to get faster answers for themselves. However, law enforcement professionals have already been forced to wear many more hats (i.e., family counselor, advocate, mediator, social worker, therapist, etc.). Should forensic analysis really be added to their hat collection when law enforcement staffing levels are struggling to keep up with basic services? Is this the most effective way to deliver forensic services? Our response is no and no - forensic laboratories already have robust quality systems and the expertise to deliver scientific results in place. Forensic scientists possess the knowledge, experience and background to expand and alter their services rather than defer responsibility for analysis to those they should be serving. Forensic results are objective, independent, based on data, and reproducible, with established acceptance in the courts. Ultimately, the options for addressing the need for speed include adding more to the plate of stressed law enforcement agency resources, adding staff that require long training programs in forensic labs, or, as we recommend, recasting the current forensic service delivery model.

## 2 Problem definition and forensic case analysis flow model

The issue can be described by the following two problem statements:

• How do “Forensic Science Service Providers” (FSSPs) better illustrate their system and the need for resources?
• Is there a better way to deliver the product of forensic leads?

A model will be provided to describe the problem statement of “How do Forensic Science Service Providers (FSSPs) better illustrate their system and the need for resources”? The airport security screening model, as employed by the United States Transportation Security Administration (TSA) will illustrate the problem definition for incoming cases through an analogy that most individuals who have travelled through American airports can easily visualize.

The airport security screening model serves as an analogy for the forensic laboratory capacity problem, with the indicator of a long line representing a backlog of cases awaiting analysis. Is the problem a long line, or is the long line an indicator of a capacity problem? Perhaps the long line is an indicator of a delivery problem. The analogy serves to illustrate the nature of the underlying issues of capacity and delivery models in providing timely forensic leads.

Traditionally in forensic laboratories, when demand increases, new priorities are set, or rush requests for testing surge, necessary resources must be reallocated from the general caseload testing pool. For example, if a laboratory was asked or mandated to prioritize one type of case above all others due to either public demand or legislative action, but with no additional resources or lagging resources, it is akin to adding another priority lane to the airport security screening checkpoint without adding any additional agents or screening equipment. The existing staffing has no choice but to redirect their efforts to address the new priorities, which inevitably allows large backups to develop quickly for other lines, even if those lines were a previous priority.

The analogy begins with a customer arriving at the airport for a scheduled flight. To get to the gate where the flight departs, the customer must pass through airport security screening. A customer is the equivalent of a case needing forensic analysis and a flight is a court case or the case's eventual resolution. There is a main line for all customers. Forensic cases are submitted to the laboratory through a standard submission process. There is also a pre-check line for higher priority customers who jump the queue but slow the main line, which are known as priority cases. There is also a line for airline and airport staff who take precedence, which are emergency exigent cases. This would be the equivalent of a very high-profile urgent case, such as the case of a 9-year-old girl abducted in New York [5] or the University of Idaho quadruple homicide when the suspect was still at large [6,7].

A relatively new line has been forming in many American states in addition to the aforementioned “priority” lines aimed at addressing newly identified priority projects set by state statutes or other stakeholder groups and external pressures. This additional priority line (similar to CLEAR in United States airports) would represent mandated testing of all sexual assault kits in a given timeframe.

If the line does not move quickly enough, the priority of the customers in the main line starts to increase as they will begin to miss flights because court cases now create a high priority. A lack of capacity causes the system to incrementally break down. Cases of lower priority do not get analyzed, uninformed investigative choices get made, cases that would benefit from forensic analysis are not submitted, such as property crimes, and the targeted solution of a forensic lead is not applied to solve cases earlier to interrupt continued criminal activity. Added to the complexity are things like weather delays and personnel issues such as a flight crew running late.

The analogy becomes clearer when considering unique case situations that, while not insurmountable, add time to the process. One customer working their way through the security line may have an infant, stroller, oversized luggage and a dog, demonstrating cases are often not one size fits all. The number of flights is increasing with increased demand for service; however the airport security screening resources are frequently fixed. This is similar to the physical plant of the forensic laboratory which can only hold so many people and instruments. Finally, all individuals must be screened as if they are boarding a plane. While it is true most customers do indeed end up on a plane, this is not true for forensic cases. Most current case processing approaches include testing to the level as if they are going to court when, in fact, only a small percentage actually do.

The airport security-screening model serves to illustrate and improve recognition of the complexity of the issues confronting forensic laboratories. Underlying causes and their solutions are not immediately clear to high-level decision makers tasked with the responsibility to provide solutions and support to the system and services. Training of screening personnel takes time and resources as does adding new lines or extra capacity. The solution we propose is to respond to this need by applying a right-sized solution. This is matching the flight customer to the correct security lane, or rather, right-sizing the case to the correct analysis lane to match the case needs to avoid slowing other cases in other lines.

## 3 Method proposal

In the description of our proposed solution, the concept of lead with speed is achieved by implementing the Forensic Lead Program (FLP). The FLP proposal provides a quick response with the benefit of technology. The result is better use of resources through utilizing a service delivery model that is right-sized through a separate product line aimed at screening.

To build on the airport security screening analogy, this proposal takes the airport security screening model and involves the addition of dedicated staffing and resources to mitigate negative impacts and support increased efficiencies for the standard case flow to continue. Further separation from the normal queue (standard airport security screening line) by establishing a separate “line” with the distinct purpose of triaging, screening, and expedited delivery of forensic leads would result in continuous and effortless integration of new programs without negatively impacting the existing caseload.

To further illustrate this new FLP model and its application to outgoing cases, we will use an analogy of restaurant meals. To connect this to the airport security screening illustration for incoming cases, the level of service of the restaurant meal is analogous to the level of security screening performed, which is equivalent to the level of forensic analysis required to provide a result or investigative lead. Investigators attend a crime scene and find evidence with forensic potential … perhaps a potato, for example. These investigators are hungry. In fact, they are starving. It is more effective to produce french fries with short order cooks to quickly satisfy investigators' hunger for a lead than it is potato croquettes with Michelin star chefs. Recognizing that french fries (fast food) are not necessarily suitable for court, a more complete preparation of the rest of the potato can be served when the customer demands it or still needs it. Without the clamor of the short order kitchen, the chefs’ smooth and methodical process allows for the delivery of a more polished product, such as potato croquettes, when the pressure for speed is less. Forensic laboratory product lines have evolved into full, sit-down meals when all that may be necessary is a quick snack.

The success of the FLP rests upon quicker implementation of targeted programs that are right-sized to the need. A fast-food meal is delivered to meet the immediate hunger with a forensic lead, followed by a full course meal as fuller analyses are needed for court. These forensic leads are also coordinated into an appetizing comprehensive meal utilizing expeditors, who oversee the process with expertise, experience and customer service focus. These expeditors would also recognize cases where there is not sufficient quantity or quality of potato to provide both french fries and a full course meal. In these instances the short order line would be skipped to provide the higher level of expertise to the limited potato. Risk assessment capability and a higher level of expertise would be critical expeditor traits.

Another consideration relevant to the delivery of forensic leads is the reporting strategy. For the information to serve in the manner in which it is designed – as actionable investigative leads – it much be organized in a consistent, concise, and understandable format. The Streamlined Forensic Reporting (SFR) initiative in England and Wales is an example of how implementing a level of consistency can be extremely beneficial when delivering results. The SFR “gives practitioners tools for a consistent approach to reporting forensic outcomes, allowing investigations and prosecutions to be progressed fairly and effectively using high-quality, relevant forensic science [8]”.

A fast-food meal requires less resources to provide, and also less resources to train those who prepare the meal. We can right-size the training to the right duty by adding a fast-food lane to provide meals that are just big enough to feed the hunger, hence the lead with speed concept. A quick screening lane can respond to the need with just enough analysis quickly (fast food meal), leaving the full-service analysis for court (full-service meal) when the time pressure of the investigation has abated.

### 3.1 Minnesota violent crime investigations support unit

One example of the FLP model in action is the recent formation of the Violent Crime Support Unit (VCSU) at the MN Bureau of Criminal Apprehension Forensic Science Services. The formal creation of this Unit was based on the successes of the statewide National Integrated Ballistics Information Network (NIBIN) program expansion project that will be summarized below.

This Unit operates under the F.I.R.S.T concept, leveraging the investigative power of Forensic Intelligence, Response, Support, and Testing. The “Forensic Intelligence” component of the F.I.R.S.T. acronym is still in development and hence should not be confused with the generation of leads through testing. Progressive forensic technologies combined with traditional forensic screening processes are prioritized to provide specialized services for violent crime investigations. This Unit was established to build upon the existing NIBIN program and to include evolving Rapid DNA protocols and the long-established Crime Scene Response program, while integrating other evidence screening protocols currently used throughout the laboratory.

The intent for consolidation and expansion of current efforts into one Unit was a more efficient delivery of expedited services, ultimately increasing the efficiency of each laboratory section through triaged testing practices. The emphasis on front-end forensic services will address the increased demand for expedited release of forensic results, primarily for violent crimes across the state. Limiting the activities of this section and its dedicated personnel allows for focused roles requiring short, targeted training. Working under the direction of an experienced program leader, newer staff can train quickly on a small number of defined tasks to provide quick forensic leads.

An added benefit of this program is that it can serve as a proving ground of sorts. It allows time to evaluate personnel performance and identify strengths and developmental needs prior to investing in a more costly and time-intensive forensic laboratory training program. Entry-level technician positions are generally great opportunities for staff to develop a familiarity with the forensic laboratory system, the general concept of evidence control, and organizational culture. Additionally, these positions, screening procedures and training programs are less costly, quickly scalable, and permit experienced forensic scientists to focus on the more complex aspects of laboratory testing.

Prior to the formation of the VCSU, the BCA NIBIN program resided within the Firearms Section as it does for many state laboratories. As demand for general firearms analysis continued to grow and staffing became more of a challenge, attention to the NIBIN entries and correlation confirmations fell in priority. To link this back to the airport security checkpoint analogy, NIBIN cases were languishing in the general line while the limited staff focused on the priority line. The resulting increase in delay meant leads were being provided well after they would have been most effective in assisting in an investigation. The cost for forensic firearm scientist experts is the largest expense and the most limited resource in providing a traditional delivery of NIBIN results. A full evaluation of firearm examiner duties was initiated to identify activities that did not require a fully trained scientist to perform. During this project, the staffing situation was targeted first, which led to several impactful changes to the NIBIN program. These included relocation of the NIBIN station from the laboratory space, training of NIBIN technicians, training of external partners to facilitate self-entry, use of the newly established ATF NIBIN Correlation Center and redefining when confirmation microscopic comparisons were performed. In other words, the delivery model was revised and the short order cook concept was applied. Investments in the program also went beyond staffing to include an expansion of the NIBIN instrument fleet to increase internal capacity while maintaining access to external partners. These changes were implemented while maintaining NIBIN database activity under the laboratory's existing accreditation. As of the writing of this report, this approach has enabled the elimination of a significant crime firearm backlog, a decrease in turn-around time from multiple months to two weeks with future improvements projected as the attention turns to evaluating further process improvements based on risk.

Regular risk assessments are necessary during the evaluation of technologies and efficiency measures to maintain the balance between speed and responsible evidence collection and testing. For example, an ongoing challenge to efficient implementation of a NIBIN program within a forensic laboratory setting has been the consideration for other forensic evidence that may be lost during the test fire and acquisition processes. This has consistently created roadblocks to meeting the aggressive (yet warranted) timeliness goals established by the Minimum Required Operating Standards for National Integrated Ballistic Information Network Sites (MROS) put forth by the ATF [9]. Evaluation of testing success rates, variability in evidence, staff training, and case impacts are measures that can inform the development of triage and testing strategies for the timely delivery of actionable investigative leads with an eye toward protecting additional forensic evidence that may be needed for future investigative and legal action.

Examples of other approaches to crime firearm analysis and triage can be found in agencies such as Miami-Dade Police Department Forensic Services Division, Philadelphia Police Department Crime Laboratory, and the Phoenix Police Department Crime Laboratory [[10], [11], [12], [13], [14], [15], [16]].

### 3.2 New York crime Firearm possession state DNA database index

On December 4th, 2023, the state of New York implemented a crime firearm possession index in its state DNA Database. The National DNA Index System (NDIS) Operational Procedures set forth by the Federal Bureau of Investigation (FBI) prohibit the use of DNA profiles from items on the person, property and home of individuals lest they be used as deduced knowns [17]. There is no such issue with this application at the state level (SDIS – State DNA Index System) with legal authority.

On pages 33–34 of the NDIS Procedures [17] it is stated:

“The Forensic Indexes contain DNA records obtained from forensic samples recovered directly from the crime scene, the victim (such as a sexual assault evidence kit, see below for additional detail), or the victim's clothing, and are attributable to the putative perpetrator. Putative perpetrator DNA recovered from the victim's body and/or clothing is crime scene evidence and is therefore eligible for upload to NDIS. Forensic unknown, forensic mixture or forensic partial DNA records from solved and unsolved cases are eligible for upload to NDIS. For cases in which the identity of the putative perpetrator is known, it is important to ensure that the DNA profile is developed from crime scene evidence and not from samples independent of the crime/crime scene.

DNA samples from known suspects that are collected independent from the crime/crime scene are considered suspect or deduced suspect DNA records; as such, they are not generally eligible for NDIS unless State law authorizes the collection and databasing of suspect samples and the FBI's CODIS Unit has approved the State to upload those as Legal DNA record(s) to NDIS.

*Special considerations for suspect DNA records*

For purposes of NDIS eligibility, an item taken directly from a suspect shall generally not be considered a forensic sample but shall be considered as a suspect or deduced suspect sample. An item on which the suspect's profile could reasonably be expected to be found that is taken from the crime scene or is part of the crime scene independent of the crime (e.g., suspect's car) are examples of suspect or deduced suspect DNA records that are not eligible for NDIS. Suspect samples also include:

• DNA samples obtained directly from a suspect;
• Items taken directly from the suspect (e.g., clothing); or
• Items in the suspect's possession (e.g., backpack being worn by suspect, suspect's home, or suspect's car).

Items of evidence collected directly from the suspect in connection with criminal possession offenses may be difficult to categorize for NDIS eligibility purposes. For example, a swabbing of a gun taken directly from the suspect or an item the suspect is wearing (e.g., clothing, backpack) is considered a deduced suspect sample and thus, is not eligible for entry into NDIS. While this evidence may be probative in proving a central element of the criminal possession offense, the sample is not a forensic unknown for NDIS eligibility and searching purposes [17].”

Experience in New York has determined that firearms often have profiles that are not that of the possessor, as firearms are frequently passed between individuals, some of whom may be collaborators. Firearms themselves may be illegal due to modifications such as switches, auto-sears, binary triggers, and privately made firearms. Firearms are also being disassembled and reassembled to commit crimes, thereby leading to DNA profiles from other contributors. Further, firearms possessors frequently hold the firearm not on their person but within their property (e.g. under the seat of the car they are driving) so it can be accessed when needed. In these situations, suspects may claim an illegally possessed firearm is not theirs, thereby necessitating DNA or other evidence to associate the firearm to the offender.

Firearms offenses are a major public safety issue. Firearms are also excellent substrates for DNA retention and transfer, with non-porous surfaces that have knurling and etching for grip. Firearms are also frequently actively handled with areas of focused contact by perpetrators directly with their skin.

The SDIS (State DNA Index System) Crime Firearm Possession Index is a New York State DNA Index established to maintain DNA records from firearm evidence collected during investigation of an unlawfully possessed firearm. The SDIS Crime Firearm Possession Index is specific to New York State, and it is among the first, if not the first statewide index of its kind in the nation. There are eight accredited forensic laboratories in New York State who can voluntarily use this index. The Index utilizes CODIS (Combined DNA Index System) software to search DNA records to generate potential investigative leads for law enforcement agencies. Based on these searches, DNA profiles developed from firearm evidence can generate associations to potential suspects while excluding other individuals as donors of DNA on a firearm. These efforts simultaneously protect individual rights while enhancing public safety.

CODIS has been highly successful in New York State in providing forensic leads to law enforcement agencies. The first 3 months in operation of the NY Crime Firearm Possession Index in February 2024, had 176 profiles uploaded to the state index by 4 voluntarily participating forensic laboratories [18]. These 176 profile uploads resulted in 43 hits, for a 24.4 % hit rate. CODIS, coupled with the success of NIBIN, provides powerful objective information to advance criminal investigations to support public safety. We expect these leads to permit law enforcement agencies to focus on reducing gun violence by removing crime firearms off the streets, to protect victims and neighborhoods in New York State, and prevent future crimes.

### 3.3 Forensic lead program

A Forensic Lead Program (FLP) establishes a permanent section which resides under the umbrella of an accredited forensic laboratory. The FLP is tasked with the development and release of forensic leads. The workflow of the FLP would parallel that described in the short order cook model, with an appropriate job classification, description and duties, training, and inclusion in a prescribed workflow to deliver screening results for forensic leads. Key features of a FLP includes a focused menu of services, narrow personnel roles, strict screening protocols, short turnaround time, standardized expectations, and targeted training. These features are combined with the sense of urgency needed for consistent delivery of timely and actionable forensic leads.

Junior forensic scientists, including those newly hired at a FSSP are made quickly functional utilizing a targeted training protocol. Their duties could include evidence screening, sample triage and preparation, and other analyses that both provide the potential for a quick lead and triage items to facilitate full service forensic analyses downstream. Experienced expeditors oversee the operation, ensuring risk is minimized and critical items are expedited for full analysis. Evidentiary items with insufficient quality and/or quantity that would be potentially compromised by screening would be sent directly for more specific analyses. Early experience with the FLP via the experience of the authors’ initiatives has indicated that investigators are very thrilled with quick responses on cases thus far as programs are moving their cases forward.

Services envisioned for the FLP include applications such as Rapid DNA technology, latent print processing, trace evidence collection, swabbing for DNA, firearms operability, creating firearms test fires, NIBIN image acquisition, utilizing handheld drug detection equipment, digital evidence collection, serial number restoration, high volume DNA collection and robotic plate preparation, and general triaging of evidence based on questions to be answered. FSSPs can adopt as few or as many as is suitable for their caseload and in iterations as supported by resources and data.

## 4 Results

Several previous business cases for forensic DNA utilize no-suspect sexual assault cases to demonstrate the value of forensic science in monetary terms [1,[19], [20], [21]]. No-suspect sexual assault cases are used because semen is a unique and very probative type of evidence to locate a suspect. It contains large amounts of DNA and is frequently very determinative as to who may have committed the crime. If a victim cannot identify their attacker, there is virtually no other mechanism to identify or eliminate the suspect. Therefore, with no other viable solution to solving these types of cases, application of forensic DNA and DNA databases is relied upon as a foundation of these business cases.

The no-suspect sexual assault forensic DNA business case model employs two factors to estimate the damages caused by crime. These are the cost of crime itself [1,21] and a recidivist factor [20,21]. The cost of the crime is composed of expenses due to the crime such as medical bills, time away from work, etc., and willingness to pay. This total cost of crime in turn is multiplied by a recidivist factor, which is the number of crimes which would be prevented if forensic technology was applied at the first opportunity, to prevent future crimes from occurring through early apprehension of the perpetrator.

The proposed firearms business case requires more assumptions, as the crime types committed by shooters vary, and other mechanisms exist which could possibly be funded to also solve the crime. These include increased law enforcement with door-to-door canvassing, shot spotters, etc. Although firearms offenses are known to be recidivist offenses with perpetrators conducting multiple crimes within short time frames [22], a specific recidivist factor for firearms offenses is not well known. Various other more traditional means can help an investigator solve the case; however, they frequently are overrun with cases and often there are no witnesses, or witnesses fail to come forward. Firearms offenses are highly serial offenses with the same individuals committing the same offense over and over, often with the same weapon, or sharing weapons among gang members or co-conspirators. Firearms offenses inflict a high level of public safety threat to society, including homicides, armed robberies, enabling other violent and serious felonies, including killing or injuring innocent bystanders.

Illegal and illegally possessed firearms represent a high risk as those prohibited from having a firearm require the power a firearm enables to commit crimes, while the illegal gun is untraceable. The damage inflicted to victims and society is high, and a solution lies in applying existing forensic technology. While a lead alone does not solve a case, it is part of a thorough investigation which must be conducted on each case prior to trial. The business case serves as an illustration of the value of the lead to move the investigation forward, not replacing the investigation. With these caveats, a business case will be applied to provide a monetized impact of the investigations impacted by the application of NIBIN for the current cases only, recognizing there is a large recidivist impact not included, as a tradeoff for the assumption these leads are pivotal in moving firearms investigations forward. The damage of crime will be estimated using the cost of each type of crime that is known for homicide, aggravated assault, armed robbery, and burglary [23] (see Table 1).

**Table 1:** Cost of crime by offense type [6].

Case type	Cost of Crime
Homicide	$17,252,656.00
Assault	$145,379.00
Robbery	$335,733.00
Burglary	$41,288.00

### 4.1 Firearms business case - DNA

New York State Police Crime Laboratory System processed 2347 swabs from firearms in 2022 and 1935 firearms swabs in 2023 as of November 13, 2023. In that time, scientists entered 28 DNA profiles into NDIS in 2022 and 9 profiles in 2023. The successful NDIS profile generation rate from firearms swabs is approximately 0.08 % (((28 + 9)/(2347 + 1935))×100). This low success rate is partially due to limits on eligibility prior to the state index implementation. Anecdotally, implementation of probabilistic genotyping is resulting in a much higher profile success rate, however statistics are not available at this time. Those 37 CODIS profiles resulted in 18 and 8 hits in 2022 and 2023 respectively. These hits provide a 64 % and 89 % hit rate for years 2022 and 2023 respectively, for a combined 70 % hit rate, which represents a very high CODIS hit rate. Previous hit rates for the United States typically average closer to 50 % for mature cold case projects [19]. The cost savings represented by these hits is provided in Table 2.

**Table 2:** Cost of crimes aided by crime firearm DNA matches 2022–2023.

Case Type	Number of CODIS Hits	Cost of Crime Type [23]	Total Cost of Cases
Homicide	4	$17,252,656	$69,010,624
Aggravated Assaults (Attempted Murder/Assault):	5	$145,379	$726,895
Robbery	**1**	$335,733	$335,733
Burglary	**1**	$41,288	$41,288
Total	**11**		$70,114,540

Discussion with other forensic laboratories indicates the expected success rate on expended cartridge cases coupling probabilistic genotyping with DNA analysis results in 6%–12 % NDIS eligible profiles [16,24]. Given that multiple cartridge cases are frequently expended in shooting cases, the success rate on a per case basis increased to 30–40 %, versus the 6–12 % result on a per item basis for expended cartridge cases [16,24].

As demonstrated in Table 2, there were a total of 11 CODIS hits that included the cases of homicide, aggravated assault, robbery and burglary. The costs of these crimes are $17,252,656, $145,379, $335,733 and $41,288 respectively [23]. The total cost of these crimes is $70,114,540.

The expense of providing this forensic DNA analysis and comparison must be calculated to determine a return on investment. Project FORESIGHT provides a median cost per sample for forensic laboratory analysis [25]. The median cost per sample of $342 for DNA casework will be utilized to account for expenses including “allocations for capital, wages & salary, benefits, overtime & temporary hires, chemicals, reagents, consumables, gases, travel, quality assurance and accreditation, subcontracting, service of instruments, advertisements, non-instrument repairs and maintenance, equipment leasing, utilities, telecommunications, overhead, and other expenses.” [25] With 2347 swabs analyzed in 2022 and 1935 swabs analyzed in 2023, the total expense of analyzing firearms swabs for DNA is $1,464,444 ((2347 + 1935) X $342)). The savings of conducting DNA on firearms is $70,114,540 with the expense of DNA analysis estimated at $1,464,444, therefore the ROI is $47.88 for every $1 spent or an 4,788 % ROI.

$70,114,540 cost of crime/$1,464,444 expense for DNA analyses = $47.88 per $1 ROI

This ROI illustration demonstrates the very sound investment in conducting forensic DNA analysis on firearms cases.

### 4.2 Firearms business case – NIBIN

As noted in the Firearms Business Case – DNA above, for the NIBIN component of the business case the cost of each type of crime that is known for homicide, aggravated assault, armed robbery, and burglary (see Table 1) [23] will be applied for the instant case only, not utilizing a recidivist factor thereby not including an estimate of savings through crime prevention. As recidivist factors are not known, they will not be included to offset the potential for cases to be solved without forensic assistance. Note that the business cases costs of crime are an illustration of the damage to individuals and society and a refund will not be due when crime rates come down, however note that as livability increases, so too does business and property value.

A tangible illustration of this cost of crime can be demonstrated using the price of property in Manhattan noting the Harlem district in the 80s and 90s when the homicide rate was approximately 2,100 annually (2,292 homicides occurred in 1990) [26]. More recently the homicide rate it has been reduced to less than 500 annually (488 in 2021 and 468 in 2020) [26] through a variety of mechanisms including greater enforcement and a ban on handguns. Therefore, the increase in property prices alone perhaps justify resident support and advocacy for additional efforts to control firearms violence, such as successful forensic laboratories supporting investigators using tools like NIBIN and DNA.

**Fig. 1:**
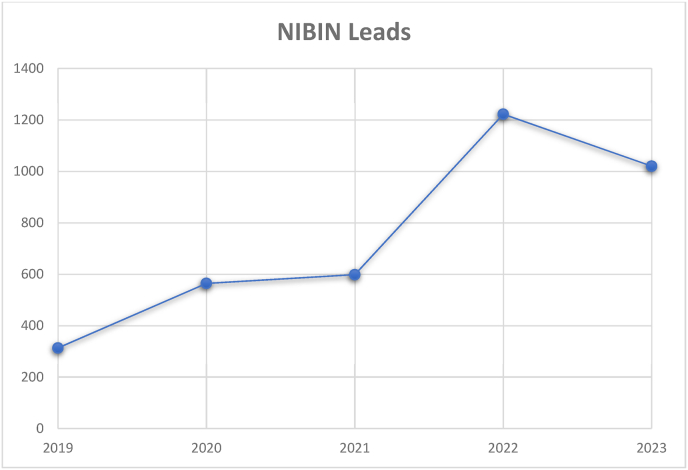
NY state police crime laboratory firearms section NIBIN leads by year.

The New York State Police Crime Laboratory System Firearms Section has been providing NIBIN leads for some time, therefore will be used as a model with leads and costs from 2019 to November 2023 (see Fig. 1).

Table 3 illustrates the NIBIN leads, cases and hit percentage by year data.

**Table 3:** NIBIN leads, cases and hit percentage by year.

Year	NIBIN Leads	NIBIN Cases	Hit Percentage
2019	313	1696	18.46 %
2020	564	1442	39.11 %
2021	598	1416	42.23 %
2022	1222	2037	59.99 %
2023	1020	1381	73.86 %
Total	3717	7972	46.63 %

Table 4 illustrates the calculation of NIBIN leads by offense utilizing Laboratory Information Management System (LIMS) data.

**Table 4:** Estimated NIBIN leads by offense type.

Offense	Percentage	Estimated NIBIN Leads
Weapons Criminal Possession	40.20 %	1494
Reckless Endangerment	34.70 %	1290
Assault	10.80 %	401
Homicide	5.50 %	204
Criminal Mischief	2.90 %	108
Criminal Possession CDS	2.70 %	100
Robbery	1.60 %	59
Burglary	1.20 %	45
Criminal Sale CDS	0.40 %	15
Total	100.00 %	3717

**Fig. 2:**
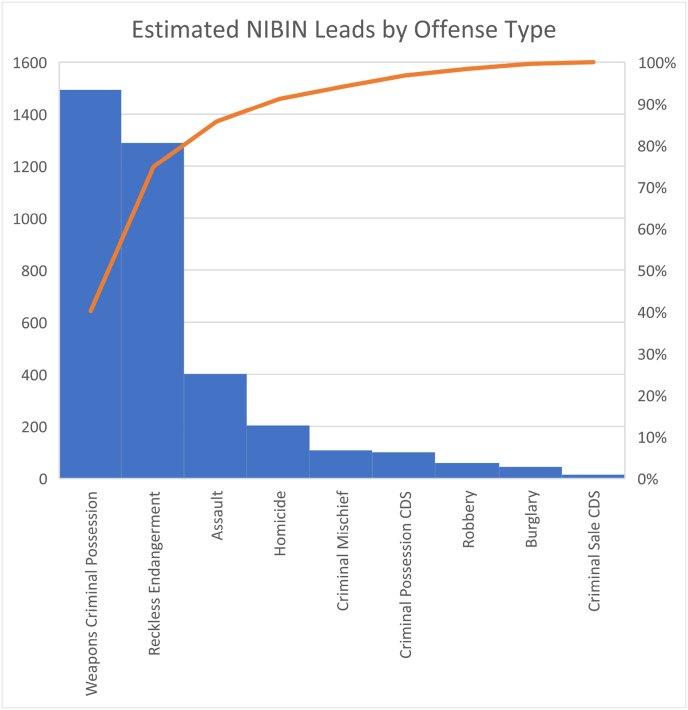
Estimated NIBIN leads by offense type.

The type of offense that the lead was matched to varies as many case types are frequently included and these types of cases are not tracked directly in LIMS. However, an estimate can be provided though the LIMS system using the percentage of NIBIN lead cases that are included in each offense type. The offense types are illustrated in Fig. 2 with the orange line indicating the total percentage of offenses.

The estimated number of offenses for each offense type was multiplied by the cost of crime for each offense type where a cost is available [23] to determine the total cost of crime involved and potentially solved by the NIBIN leads (see Table 5).

**Table 5:** Calculation of the cost of crime potentially solved by NIBIN leads.

Case type	Cost of Crime	Estimated NIBIN Leads	Cost of Crime
Homicide	$17,252,656.00	204	$3,519,541,824.00
Assault	$145,379.00	401	$58,296,979.00
Robbery	$335,733.00	59	$19,808,247.00
Burglary	$41,288.00	45	$1,857,960.00
Total cost of crime			$3,599,505,010.00

The total cost of crime potentially solved by NIBIN leads is over $3.599 Billion (see Table 5).

The next step is determining the expense of providing the NIBIN forensic laboratory service which produced these leads.

The cost for forensic firearm scientist experts is the largest expense for providing a NIBIN service. These firearms experts do far more than NIBIN work, however, to be very conservative with regards to the ROI, the entire cost of their salary and benefits will be included in the expense calculation. The indirect cost for each employee is estimated at 46 %. The top annual salary for the Forensic Scientist 3 is $88,721. This equals $129,532.66 when the indirect costs are added. With 12 forensic firearms scientist experts working in the Firearms section currently, their total cost is $6,217,567.68 (see Table 6). There are also 7 instruments used for NIBIN leads, which are 3 BRASSTRAX and 4 MatchPoint instruments, priced at approximately $130,000 and $60,000 each respectively. Also included is 1 stereomicroscope, which is approximately $20,000, and 4 comparison microscopes, which are approximately $75,000 each. The total cost of providing the NIBIN leads is therefore approximately $7,167,567.68 (see Table 6).

**Table 6:** Summary of firearms program cost.

Forensic Expenses	Cost per unit	Units	Total Cost
BRASSTRAX	$130,000.00	3	$390,000.00
MatchPoint	$60,000.00	4	$240,000.00
Comparison microscope	$75,000.00	4	$300,000.00
Stereomicroscope	$20,000.00	1	$20,000.00
Forensic Scientists	$129,532.66	48	$6,217,567.68
Total Cost			$7,167,567.68

The cost of providing the NIBIN forensic service for the last 4 years is estimated at $7,167,567.68. As the cost of crime potentially solved by NIBIN leads is $3.599 Billion and the cost of providing the service is $7,167,567.68, the ROI is $502.19 for each $1 spent. This equates to a 50,219 % ROI.

$3,599,505,010 cost of crime/$7,167,567.68 expense of NIBIN analyses = $502.19 per $1 ROI

The median expense per Firearms Database (including NIBIN) case from project FORESIGHT is $223 [25]. With a total expense of the Firearms Section labor and specific costs relating to the NIBIN program of $7,167,567.68, a total of 3,717 leads were produced. This equals $1,928 per NIBIN lead ($7,167,567.68/3717 leads) and $2,204.75 per NIBIN case ($7,167,567.68/7972 cases) when applying New York expense data. Therefore, the higher expense of $7,167,567.68 was utilized, as it yields a lower and more conservative ROI. The FORESIGHT median cost of $223 per case would provide a total cost of $1,777,756 for the 7972 NIBIN cases analyzed ($223 × 7972 cases), which would yield a much higher ROI of $2,204.75 per $1 spent ($3,599,505,010/$1,777,756).

This illustration demonstrates there is tremendously strong support for investing in NIBIN to solve and prevent firearms crime.

## 5 Discussion

It is estimated that DNA profiles from Rapid DNA instruments from single source samples found at crime scenes will become available for uploading and searching using CODIS early in 2025 [27]. Initially, forensic samples will require review by qualified DNA analysts in the accredited state CODIS laboratory. Rapid DNA is capable of generating a DNA profile from single source samples in less than 2 hours, which will enable the provision of a speedy lead for non-complex samples (no mixtures) with instruments that can be located outside of a physical forensic laboratory [2]. While there will be temptation to assign this new duty to existing crime scene units, this new responsibility will come with additional training and quality assurance requirements that fall under the scope of the currently accredited state CODIS forensic laboratory. For access to CODIS and the offender, arrestee and forensic profiles it permits for comparison, law enforcement must partner with an accredited forensic laboratory. The FLP model proposed creates the opportunity for a thoughtful design and deployment of this service.

For single source DNA samples, which are far less complex than DNA mixtures to interpret, the reduction in complexity enables a speedier analysis to provide an investigative lead. This time savings translates into a significant return on investment in cost of crime savings by preventing future crimes through solving current crimes more quickly. The typical forensic DNA scientist is capable of producing approximately 102 cases per year, which result in 24.6 CODIS leads per year [1]. These 24.6 annual leads translate to approximately 0.1119 CODIS hits per day per analyst, which saves $48,723 of cost of crime [1] daily. With recidivist factors for the model using sexual assaults ranging between 7 and 26.22 crimes solved for each DNA hit are applied, this savings escalates to a savings range from $87,000 to $1.2 million per analyst-day, which now accounts for the crimes that can be prevented by solving the instant crime more quickly [1,2]. This cost savings per analyst-day translates to a very large savings of $16,77.75 per $1 spent, or a 167,775 % ROI. These data demonstrate that the quicker leads can be generated, the cost associated with prevented crimes can be saved. Therefore, providing objective forensic leads are a mechanism to support public safety and justice, while guarding against wrongful conviction.

Firearms represent rich substrates for touch or trace DNA. As weapons are used to commit crimes, they are frequently handled directly by perpetrators with skin contact. Firearms are often possessed illegally by felons, who use them to commit additional crimes on new victims. CODIS, NIBIN and latent fingerprint databases are recidivist databases, relying on the repetitive nature of crime to provide investigative leads. A local investment in forensic science infrastructure enables access to and reward from the much larger investment of the databases. In the case of CODIS, the national database may represent a $100 million investment (20,000,000 plus offenders at $50 per profile [19]), however the return from local use more than pays for itself while justifying the larger infrastructure. This is amply demonstrated by the projected $47.88 per $1 spent ROI for forensic DNA on Firearms and $502.19 per $1 spent ROI for NIBIN participation.

As demonstrated by the firearms DNA and NIBIN business cases, there is a very large ROI by providing forensic analysis, even when calculated conservatively. Analysis of firearms evidence occurring at crime scenes permits jurisdictions to take advantage of the very large investment of CODIS and the NIBIN database. Queries can not only link crimes together, but also to a potential perpetrator. Rapid DNA and NIBIN provide the capability to apply scientific screening tests to evidence outside of a traditional forensic laboratory setting, both in terms of location and in structure of the service delivery. Forensic laboratories are well acquainted with a two-tier analysis strategy with the first step of screening followed by the second step of confirmation [2]. In the FLP model, the focus is on quick delivery of screening through an abbreviated delivery process to enable investigations to move forward.

The two examples provided have originated from state forensic laboratory systems in New York and Minnesota, which represent only one forensic laboratory type in the spectrum of United States forensic laboratories. These laboratories include federal, state, local FSSPs, which vary greatly in size, structure and forensic service offerings. Statewide systems may be better funded than local laboratories, however the larger jurisdictional size and multi-jurisdictional purview creates its own challenges which frequently includes resistance to change. This challenge can be demonstrated in a small way by a simple NIBIN metric of importance which is “Seizure to Acquisition Average”. During CY23, this average was approximately 2.5× longer for state laboratories than for individual police departments.^1^ At the same time, it is the responsibility of well-resourced labs to use their ‘privilege’ to develop and evaluate models in an effort to establish best practices. By highlighting successful ways this can be accomplished, the examples can serve to inform laboratory services of all sizes and scope.

An additional consideration specific to impacting social justice, firearms violence has a major negative impact on the neighborhoods in which it occurs. Over 80 percent of crimes are committed on the same socioeconomic group [19,28,29]. These crimes impact those most vulnerable in society, negatively impacting not only the residents’ safety and security, but also their property value as noted above in the Manhattan, NY, example. Objective evidence provided by forensic analysis is also unbiased and based on data, and capable of eliminating suspects immediately if samples do not match. As these crimes are unsolved until a suspect is developed through an objective forensic investigative lead, these unknown perpetrators are still at large available to commit additional crimes.

“One of the features of law enforcement known DNA databases which is viewed negatively is the racial and socioeconomic makeup of convicted and arrested individuals that populate them. It is very noteworthy that victims of crime live in the very neighborhoods and demographic of those committing the crimes and more likely in the same racial and socioeconomic group [28,29]. Therefore, not including individuals as potential suspects based on the background of the suspect disproportionally negatively impacts victims of the same demographic.” [19,28,29] “About half of homicides are known to be single-offender/single-victim, and most of those were interracial; in those where the perpetrator's and victim's races were known, 81 % of white victims were killed by whites and 91 % of black or African American victims were killed by blacks or African Americans.” [30,31]. Solving and preventing crime is first and foremost a public safety issue. Improved implementation and speed of forensic leads applies to crimes that have multiple dangers. Innocent victims and their families are impacted by firearms violence. Wrongfully accused individuals must be ruled out as quickly as possible. Recidivist crimes can be prevented, particularly when powerful, proven, forensic tools are now available.

Forensic scientists do not determine guilt or innocence. This is the duty of the finder of fact, which in the United States is the jury or the judge in cases where the defendant has chosen to be tried by a judge. The nature of the investigative lead is to provide investigators objective evidence to move their investigations forward. A full investigation is required rather than relying solely on forensic evidence of association, as there may be reasons unrelated to the crime that an individual may be associated with a victim or crime scene.

## 6 Summary

Forensic laboratories and law enforcement work closely together to determine which analyses are conducted on each case; however, an offering of full-service analyses does not leverage the capability of new screening techniques. With mindful planning, implementation, execution and adjustment, providing expedited leads is much more achievable with a specialized unit rather than within the existing forensic laboratory analysis model. Matching the product line with need, evidence limitations and testing capability results in a pared down but effective analytical service delivery model. Avoiding over-delivery on every item assists with overall resource preservation as the extra work now negated does not come at the expense of the next case in line. Better service is achieved with coordination of laboratory analyses and results and follow up using an experienced forensic expeditor. The result is a two-tiered approach that incorporates abbreviated analyses on less complex samples to deliver quick forensic leads, supported with full-service analyses for court.

A business case demonstrates that each additional day saved by reduced training, implementation and response time saves between $87,000 and $1.2 Million [1]. NIBIN hit rates of over 100 % are being achieved as the number of investigations aided are surpassing the number of cases being investigated within the authors’ forensic laboratories, due to the frequency of leads including multiple case linkages. DNA and CODIS results with 24.4 % and 70 % hit rates for each crime firearm entered have been achived. Results from forensic laboratories demonstrate the cost savings of $47.88 per $1 spent, which is a 4,788 % return on investment. The return on investment of NIBIN is $502.19 for each $1 spent. This equates to a 50,219 % return on investment.

All travelers would agree that a great start to any trip would involve a quick flowing security screening line, free from unexpected delays, bottlenecks, and other people cutting in line. Everyone appreciates there are situations that require certain people to move to the front of the line. However, it would certainly be less frustrating for those situations to be addressed in a completely separate line to avoid disrupting the efficient flow of the main line. Forensic lead testing as a standalone program would serve in this same manner.

Currently available technology permits a more effective forensic analytical response than what is currently being provided routinely by forensic laboratories. Demand for analysis is continuously outstripping demand, resulting in backlogs and delayed response times. Speed for forensic leads is critical to eliminate and include suspects when resources are being expended. The business case readily demonstrates the proposed model is well supported. It is time to recast the product line and service delivery model to better fit the need for speed. This requires resources, planning and coordination along with an appreciation for the unique and, at times, fluid needs of the criminal justice community. This will allow for the right-sized delivery of impactful and responsive forensic leads. The proposed Forensic Lead Program enables forensic laboratories to lead with speed.

## CRediT authorship contribution statement

**Ray A. Wickenheiser:** Writing – review & editing, Writing – original draft, Visualization, Project administration, Methodology, Investigation, Formal analysis, Data curation, Conceptualization. **Catherine M. Knutson:** Writing – review & editing, Writing – original draft, Visualization, Project administration, Methodology, Investigation, Funding acquisition, Formal analysis, Conceptualization.

## Declaration of competing interest

The authors declare that they have no known competing financial interests or personal relationships that could have appeared to influence the work reported in this paper.

## Footnotes

1 Data reported in the NIBIN FY24 December 2023 Monthly Activity Report provided by the ATF to NIBIN sites was used to calculate this number.
